# Correlation of senescence-related gene FEN1 on neuroblastoma progression and cisplatin chemotherapy sensitivity

**DOI:** 10.32604/or.2025.060021

**Published:** 2025-06-26

**Authors:** YOUYANG HU, YISHU LUO, TIANYUE XIE, YUEHUA CHEN, JUN ZHAO, WEICHAO JI, ZHIWEI YAN, SITONG QIU, KEXIN GAO, HAIXIA ZHU, LIMIN MA, QIYOU YIN

**Affiliations:** 1 Pediatric Surgery, Affiliated Hospital of Nantong University, Nantong, 226001, China; 2 Pediatric Surgery, Children’s Hospital of Fudan University, Shanghai, 201100, China; 3 Pediatric Surgery, Medical College of Nantong University, Nantong, 226001, China; 4 Cancer Research Center Nantong, Affiliated Tumor Hospital of Nantong University & Nantong Tumor Hospital, Nantong, 226361, China

**Keywords:** Flap endonuclease-1 (FEN1), Cellular senescence, Neuroblastoma (NB), Chemotherapy, IC50, Prognostic model

## Abstract

**Objective:**

Neuroblastoma (NB) is frequently associated with high-risk pediatric cases that demonstrate limited response to cisplatin, contributing to a poor prognosis. Recent studies have explored the role of tumor cell senescence in increasing sensitivity to this chemotherapy agent. This study aims to identify genes related to cell senescence in children diagnosed with NB, evaluate their influence on cisplatin sensitivity, and investigate potential strategies to enhance the efficacy of chemotherapy.

**Methods:**

Gene expression profiles and clinical data were obtained for 498 NB patients from the GEO database (GSE49710). The study focused on identifying genes that were differentially expressed between stage IV and other stages, particularly those linked to cell senescence and cisplatin resistance. To analyze the prognostic significance of these differentially expressed genes, we employed LASSO regression and multivariate Cox proportional hazards models. Transcriptomic and proteomic analyses of 15 NB specimens revealed a significant correlation between Flap endonuclease-1 (FEN1) expression levels and both cellular senescence and sensitivity to cisplatin. We quantified FEN1 expression and cisplatin IC50 values in four different NB cell lines. The influence of FEN1 knockdown and overexpression on NB cell proliferation, invasion, and migration was evaluated using cloning assays, transwell assays, and scratch assays. Furthermore, we utilized Western blotting to analyze senescence-associated proteins p21 and proliferating cell nuclear antigen (PCNA), thereby evaluating the role of FEN1 in cellular senescence. The impact of FEN1 on cisplatin sensitivity was investigated via the CCK-8 cell counting assay. Additionally, we investigated how FEN1 inhibitors might impact NB cell proliferation and enhance the therapeutic efficacy of cisplatin treatment.

**Results:**

FEN1 was found to be highly expressed in stage IV NB and showed a strong association with cisplatin sensitivity, establishing it as a critical molecular marker linked to poor patient prognosis. Notably, elevated FEN1 expression correlated with reduced sensitivity to cisplatin, as evidenced by higher IC50 values. In the SH-SY5Y cell line, FEN1 knockdown led to significant reductions in cell proliferation, invasion, and migration, along with an increase in β-galactosidase staining—indicative of senescence. This knockdown also resulted in elevated levels of the p21 protein and decreased expression of PCNA, concurrently lowering cisplatin IC50 values. Conversely, FEN1 overexpression in the SK-N-SH cell line resulted in enhanced cell proliferation, invasion, and migration. This overexpression was associated with reduced β-galactosidase staining, decreased levels of p21, and increased expression of PCNA, ultimately resulting in higher cisplatin IC50 values. Importantly, FEN1 inhibitors alone significantly impeded NB cell proliferation, and their combination with cisplatin further amplified this inhibitory effect compared to cisplatin treatment alone.

**Conclusions:**

Bioinformatics and sequencing analyses indicate that the senescence-related gene FEN1 is significantly associated with cisplatin sensitivity and adverse prognosis in pediatric NB. FEN1 plays a pivotal role in regulating NB cell proliferation, invasion, and migration, thereby facilitating cancer progression. Furthermore, it influences cisplatin sensitivity through its effects on cellular senescence. FEN1 inhibitors demonstrate potential both as monotherapies and in conjunction with cisplatin, suggesting that targeting FEN1 may be represent a valuable strategy for improving outcomes in high-risk NB patients.

## Introduction

Neuroblastoma (NB) is the most prevalent extracranial solid tumor in pediatric patients, often characterized by aggressive progression [1]. Treatment strategies for NB are dictated by risk stratification: surgical intervention is generally recommended for low-risk patients, whereas high-risk patients typically undergo chemotherapy [2]. Cisplatin is a commonly utilized agent in these chemotherapy regimens and has been shown to decrease mortality rates [3]. However, prolonged exposure to cisplatin can lead to diminished drug sensitivity, resulting in tumor recurrence, poor prognosis, and increased mortality. The decline in drug sensitivity is a critical contributor to the unfavorable outcomes observed in high-risk NB patients. Nevertheless,, the molecular mechanisms underlying this reduction in sensitivity warrant further investigation.

Cellular senescence refers to the irreversible cessation of cell proliferation, characterized by cell cycle arrest, structural alterations, and reduced functionality [4]. Senescent cells exhibit features such as genomic instability, telomere shortening, and mitochondrial dysfunction. They also release a variety of factors known as the senescence-associated secretory phenotype (SASP), which can recruit immune cells and potentially impede tumor progression [5–7].

In addition to curtailing the indefinite proliferation of tumor cells, cellular senescence may also increase chemotherapy sensitivity. For instance, Ge et al. showed that dexamethasone reduced the expression of the senescence-related protein p53, leading to decreased cisplatin sensitivity in patients with non-small cell lung cancer [8]. Similarly, Álvarez-Abril et al. found that inducing cellular senescence in mouse models of ovarian cancer activated immune effector cells, which enhanced cisplatin sensitivity [9]. Pandey et al. reported that inducing senescence in breast cancer cells could mitigate overcome cisplatin resistance [10]. Conversely, Sun et al. discovered that inhibiting cellular senescence in ovarian cancer cells promoted cisplatin resistance [11]. Additionally, Nakayama and colleagues observed that upregulation of NAC1 in ovarian cancer cells suppressed cellular senescence and reduced cisplatin sensitivity [12]. Collectively, these studies underscore that inducing cellular senescence can improve the efficacy of cisplatin across various cancer types [13]. However, there is a paucity of research focusing on the mechanisms of cellular senescence in pediatric NB. Therefore, this study aims to establish a solid laboratory foundation for managing high-risk NB patients by investigating key genes implicated in NB cell senescence and their effects on the chemotherapeutic response to cisplatin.

Flap endonuclease-1 (FEN1) plays a pivotal role in the base excision repair (BER) pathway, which is vital for DNA repair, processing Okazaki fragments during DNA replication, and regulating other DNA repair mechanisms such as homologous recombination repair (HRR) and non-homologous end joining (NHEJ) [14]. The expression of FEN1 is often upregulated in various solid tumors, including ovarian, breast, and non-small cell lung cancers, contributing to enhanced tumor progression and chemotherapy resistance [15–17]. Elevated levels of FEN1 are associated with increased metastasis and unfavorable clinical outcomes [18,19].

Research has indicated that modulating FEN1 expression can alter sensitivity to cisplatin in both breast and gastric cancers [20,21]. Additionally, Xu et al. demonstrated that upregulation of FEN1 diminishes the efficacy of tamoxifen in breast cancer patients [16]. These findings underscore the potential of FEN1 as a promising therapeutic target in cancer treatment.

FEN1 is an overexpressed gene in various cancers and has been linked to poor prognosis due to its role as a senescence-related gene in tumor tissues [22,23]. Research conducted by Maremanda et al. has highlighted FEN1’s involvement in exacerbating chronic obstructive pulmonary disease (COPD) and other pulmonary conditions, attributing its effects to its direct association with cellular senescence [24]. Cisplatin, a first-line chemotherapy agent, induces tumor cell proliferation inhibition primarily through DNA damage, which serves as a key trigger for cellular senescence [25,26]. In this context, FEN1 acts as a protease that facilitates the repair of damaged DNA, thus playing a critical role in mediating the effects of cisplatin on tumor cells [27].

In this study, we propose the hypothesis that FEN1 expression significantly influences cellular senescence in NB cells and affects the efficacy of cisplatin chemotherapy. This hypothesis is supported by bioinformatics analyses of the GSE49710 and GSE86842 datasets, the GEO database, and sequencing data obtained from 15 clinical specimens of NB. The objective of this research is to investigate the role of FEN1 in mediating cellular senescence and cisplatin sensitivity in NB cells. Additionally, we aim to assess the potential therapeutic benefits of combining cisplatin with FEN1 inhibitors to enhance treatment efficacy in high-risk NB patients.

## Methods and Materials

### Data retrieval and gene expression analysis

The gene expression profiles and clinical data from the GSE49710 dataset were extracted from the GEO database (http://www.ncbi.nlm.nih.gov/geo/) (accessed on 30 January 2025). Tumor specimens were categorized into stage IV and other stages according to tumor staging guidelines. Differential analysis, conducted using the Sanger online platform (vip.sangerbox.com/index.html) (accessed on 30 January 2025), identified a total of 7062 upregulated genes and 2715 downregulated genes. Additionally, a systematic analysis was executed on 1349 genes using GEO to identify those associated with cisplatin susceptibility. To elucidate the association between cisplatin resistance and gene expression, the cisplatin resistance dataset GSE86842 was juxtaposed with the GSE49710 dataset through Venn diagram analysis to identify overlapping genes. This methodology facilitates a more profound comprehension of the genetic determinants influencing cisplatin sensitivity and resistance in NB.

### Functional enrichment analysis

Gene set enrichment analysis was conducted utilizing the KEGG REST API for current KEGG Pathway annotations, in conjunction with the org.Hs.eg.db gene annotation via the R package (version 3.1.0). The analysis utilized the clusterProfiler package (version 3.14.3) with a gene set size criterion ranging from 5 to 5000, applying a *p*-value cutoff of <0.05 to identify significant enrichment in cellular senescence pathways. Additionally, from The Ageing Gene Database (https://genomics.senescence.info/genes/index.html) (accessed on 30 January 2025), a total of 307 aging-related genes were identified. Venn diagram analysis was utilized to determine the intersection between these aging-related genes and the 1349 genes associated with cisplatin susceptibility, revealing a total of 23 overlapping genes. This overlap suggests a potential link between cellular senescence and cisplatin sensitivity, necessitating further exploration into the roles of these intersecting genes in NB.

### Survival analysis and prognostic modeling

Utilizing the R survival package (version 3.7-0), survival duration, status, and gene expression data were analyzed to identify genes critical for the prognosis of NB. The analysis employed LASSO regression and multivariate Cox models to assess the impact of gene expression on survival outcomes. The Significance of the identified genes was evaluated using Kaplan-Meier curves, and the best model yielded a lambda value of 0.107019607286109. Additionally, Receiver Operating Characteristic (ROC) analysis was performed using the pROC package (version 1.17.0.1) to estimate the area under the curve (AUC) for three key genes at various time points: 365, 1095, and 1825 days. This analysis provided insight into the predictive power of these genes for patient survival in NB, underscoring their potential relevance as prognostic biomarkers.

### Western blot protocol

Place the treated cell culture dishes on an ice box, add freshly prepared protein lysis buffer which is prepared by mixing 1 mL of RIPA strong lysis buffer (ABclonal, PC101, Shanghai, China), 20 µL of phosphatase inhibitor (ABclonal, GRF101), 10 µL of PMSF (ABclonal, ST506) and 200 µL of 5× Loading Buffer (ABclonal, LT101S). Use a cell scraper to scrape off the cellular proteins and collect them into EP tubes. Prepare resolving gel according to the instructions. Run the protein samples at a constant voltage of 80V. Prepare the transfer buffer. Pre-activate the polyvinylidene difluoride membrane (PVDF, MerckMilipore, IPVH00010) by soaking it in methanol for later use. Perform wet transfer for protein transfer. Prepare the blocking solution (ABclonal, PS108p, Shanghai, China). Block the membrane at room temperature for 2 h. Then dilute the primary antibody in an appropriate antibody diluent to the specified concentration. Place the membrane in the refrigerator at 4°C to incubate overnight. Primary antibodies used in this research are listed here: anti-FEN1(1:5000, ab109132, Abcam, Cambridge, MA, USA); anti-P21 (1:1000, ab109520, Abcam); anti-PCNA (1:1000, ab29, Abcam); anti-tubulin (1:1000, ab78078, Abcam); anti-GAPDH (1:1000, #2118, Cell Signaling Technology, Danvers, MA, USA). Then, prepare the secondary antibody by diluting it to the appropriate concentration that corresponds to the primary antibody. Incubate at room temperature for 2 h. Finally, wash the membrane again three times with TBST (ABclonal, PS103s, Shanghai, China). The following secondary antibodies were used: HRP-conjugated Goat Anti-Rabbit IgG (1:5000, SA00001-1, proteintech, Wuhan, China); HRP-conjugated Goat Anti-Rabbit IgG (1:1000, SA00001-2, proteintech). Prepare the appropriate amount of detection solution by mixing the developers, ensuring thorough mixing while avoiding exposure to light.

### Cell culture

Fifteen NB samples from Children’s Hospital of Fudan University were subjected to transcriptomic and proteomic analysis by Digital Spectrum (Shanghai, China). For cell-based experiments, four NB cell lines—SH-SY5Y, IMR-32, SK-N-AS, and SK-N-SH—were cultured Dulbecco’s Modified Eagle Medium (DMEM, Thermo Fisher Scientific Inc, C11995500BT, Waltham, MA, USA) at 37°C with 5% CO_2_. The culture medium consisted of 10% fetal bovine serum (Wuhan Elabscience Biotechnology Co., Ltd., 164210, Wuhan, China), 89% DMEM, and 1% penicillin/streptomycin (New Cell ＆ Molecular Biotech Co., Ltd., CB010, Suzhou, China). Once the cells reached 80%–90% confluence, they were harvested by trypsinization, neutralized with fresh media, and subjected to centrifugation. During the process of cell culture, it is essential to adhere strictly to the aseptic operation protocols. Regular and meticulous inspection of the cell status should be carried out. Additionally, the concentration of penicillin/streptomycin can be suitably elevated as a preventive measure against mycoplasma contamination. The resultant cell pellet was resuspended in a cryopreservation solution, transferred into cryotubes, and subsequently stored at −80°C for future experiments. This methodology ensures that the cell lines remain viable for subsequent assays and analyses.

### Cryopreservation and thawing

Cryovials containing the NB cell lines were thawed by placing them in a 37°C water bath until fully liquefied. Following the thawing process, the cells were centrifuged at 1200 rpm (Anhui USTC Zonkia Scientific Instruments Co., Ltd., SC-3616, Hefei, China) for 6 min to pellet them. The supernatant was discarded, and fresh complete DMEM medium was added to the cell pellet. The resulting cell suspension was then transferred to culture dishes and incubated at 37°C with 5% CO_2_ for optimal growth conditions. This procedure facilitates proper cell recovery and prepares them for subsequent experimental applications.

### Lentiviral transduction and selection

NB cells were prepared by digestion and centrifugation, after which they were seeded at a density of 100,000 cells per well in 6-well plates. Following a 24-h incubation period to facilitate cell adhesion, varying concentrations of lentivirus (Shanghai Genechem Co., Ltd., Shanghai, China) and an infection enhancement solution were added in accordance with the established protocol guidelines. The sequence of negative control sh-RNA is TTCTCCGAACGTGTCACGT, and the sequence of sh-RNA is GCAGTGACTACTGTGAGAGTA. After an additional 24 h, the culture medium was replaced to eliminate excess lentivirus, and puromycin was applied 72 h post-infection to select for the successfully transduced cells. This selection process ensures that only the cells expressing the lentiviral construct remain viable and continue to proliferate for downstream applications.

### Colony formation assay

Transfected NB cells were cultured at a density of 500 cells per well in 6-well plates. After allowing sufficient time for colony formation, the cells were washed with phosphate-buffered saline (PBS) to eliminate any residual medium. The cells were then fixed with 4% paraformaldehyde to preserve cell structure. Once fixed, the cells were stained with crystal violet to visualize the colonies. The staining procedure enhances the contrast, facilitating the identification and enumeration of the colonies formed. Following staining, the wells were documented photographically to capture images of the colonies, which can be used for subsequent analysis of colony formation efficiency, reflecting the growth and proliferation potential of the transfected NB cells.

### Invasion assays

Prepare a transwell insert with an appropriate pore size. Then, suspend 100,000 NB cells in serum-free medium and add 200 µL of the cell suspension to the upper chamber of the transwell. Place the transwell in a 24-well plate filled with medium in the lower chamber. Incubate the plate at 37°C with 5% CO_2_ for 24 h. The experimental design consisted of both control and experimental groups. Following the incubation period, the cells were fixed with 4% paraformaldehyde to preserve cellular structure for subsequent analysis. The cells were then stained with crystal violet to visualize the migrated or invasive cells. After staining, images of the wells were captured and analyzed using ImageJ software (National Institutes of Health, version 1.5, Bethesda, MD, USA), enabling quantification of stained cells, thereby assisting in the assessment of cell migration or invasion capabilities of the NB cells under different experimental conditions.

### Scratch assay

NB cells were cultured in 6-well plates at a density of 800,000 cells per well during normal and logarithmic growth phases. Once the cells reached over 90% confluence, a scratch was introduced into the cell layer using a 200 μL pipette tip to induce a wound healing effect. Images were captured at time points of 0, 24, 36, and 48 h post-scratch to monitor the migration of cells into the wounded area. These images were analyzed using ImageJ software, which facilitates precise quantification of the area covered by migrating cells over time. This scratch assay provides valuable insights into the migratory capacity of NB cells and can be employed to evaluate the effects of various treatments or genetic modifications on cell motility.

### Cell viability assay

Cells in both logarithmic and normal growth phases were seeded at a density of 5000 cells per well in a 96-well plate, with five replicates for each condition to ensure statistical reliability. Following the seeding, CCK-8 reagent (10 μL, ABclonal, cx001M, Shanghai, China) was added to each well at designated intervals of 0, 24, 48, and 72 h post-seeding. After allowing the CCK-8 reagent to react for a specified duration, the absorbance of each well was measured at 450 nm using a microplate reader (Thermo Fisher Scientific Inc., FL, Waltham, MA, USA). This assay assesses cell viability and proliferation, as the absorbance is proportional to the number of viable cells in each well. The data collected can be used to evaluate the growth patterns of NB cells under different conditions and time points.

### Senescence assay

About 100,000 NB cells were cultured in 6-well plates for 24 h to facilitate adequate adhesion. After this incubation period, the cells were washed with phosphate-buffered saline (PBS) to remove any residual medium. They were then fixed with β-galactosidase fixative (Beyotime Biotech Inc., C0602, Shanghai, China) for 20 min at room temperature to preserve cellular structures and prepare them for staining. Following fixation, the cells were stained with a β-galactosidase staining solution. The plates were then placed in a CO_2_-free environment and incubated overnight at 37°C to optimize staining conditions. After incubation, the cells were examined under a microscope (Leica Biosystems Nussloch GmbH, TS2-FL, Shanghai, China) to identify senescent cells, which typically exhibit characteristic blue staining due to the presence of β-galactosidase activity. This method facilitates the visualization and assessment of cellular senescence in the cultured NB cells.

### Cisplatin sensitivity assay

3000 cells were allocated into 96-well plates, with five wells designated for each experimental group corresponding to varying concentrations of cisplatin ranging from 500 to 2000 ng/mL. After a 48-h incubation period to allow for drug exposure and cellular response, 10 uL of CCK-8 reagent was added to each well. The study about the combined application of FEN1 small molecule inhibitor C20 and cisplatin set the concentration of C20 at 5 μM, the concentration of cisplatin at 1500 ng/mL, and the action time at 24 h. After an appropriate incubation time for the CCK-8 reagent to react, the absorbance was measured at 450 nm using a microplate reader. The optical density (OD450) values obtained reflect the viability of the NB cells in response to the different concentrations of cisplatin. This assay helps evaluate the cytotoxic effects of cisplatin on NB cells, providing insights into the drug’s efficacy at various concentrations.

### Data analysis

Data analyses were conducted using SPSS version 25.0 (Statistical Product and Service Solutions, YSA). The chi-square test was employed to evaluate correlations between FEN1 expression and patient demographic factors such as age, sex, and grade. To assess the prognostic significance of FEN1 expression, Cox regression analysis was performed. For data visualization, GraphPad Prism 9.0 (GraphPad Software, Inc., San Diego, CA, USA) was utilized to create histograms, line charts, and graphs for various assays, including Transwell assays, scratch assays, CCK-8 assays, and cisplatin IC50 response curves. Statistical significance was determined using paired or unpaired *t*-tests, with a significance threshold set at *p* < 0.05. This comprehensive analytical approach allows for a thorough evaluation of the relationship between FEN1 expression and clinical outcomes, as well as the effects of different treatments on NB cell viability and behavior.

## Results

### Genes associated with cisplatin sensitivity in NB

**Figure 1:**
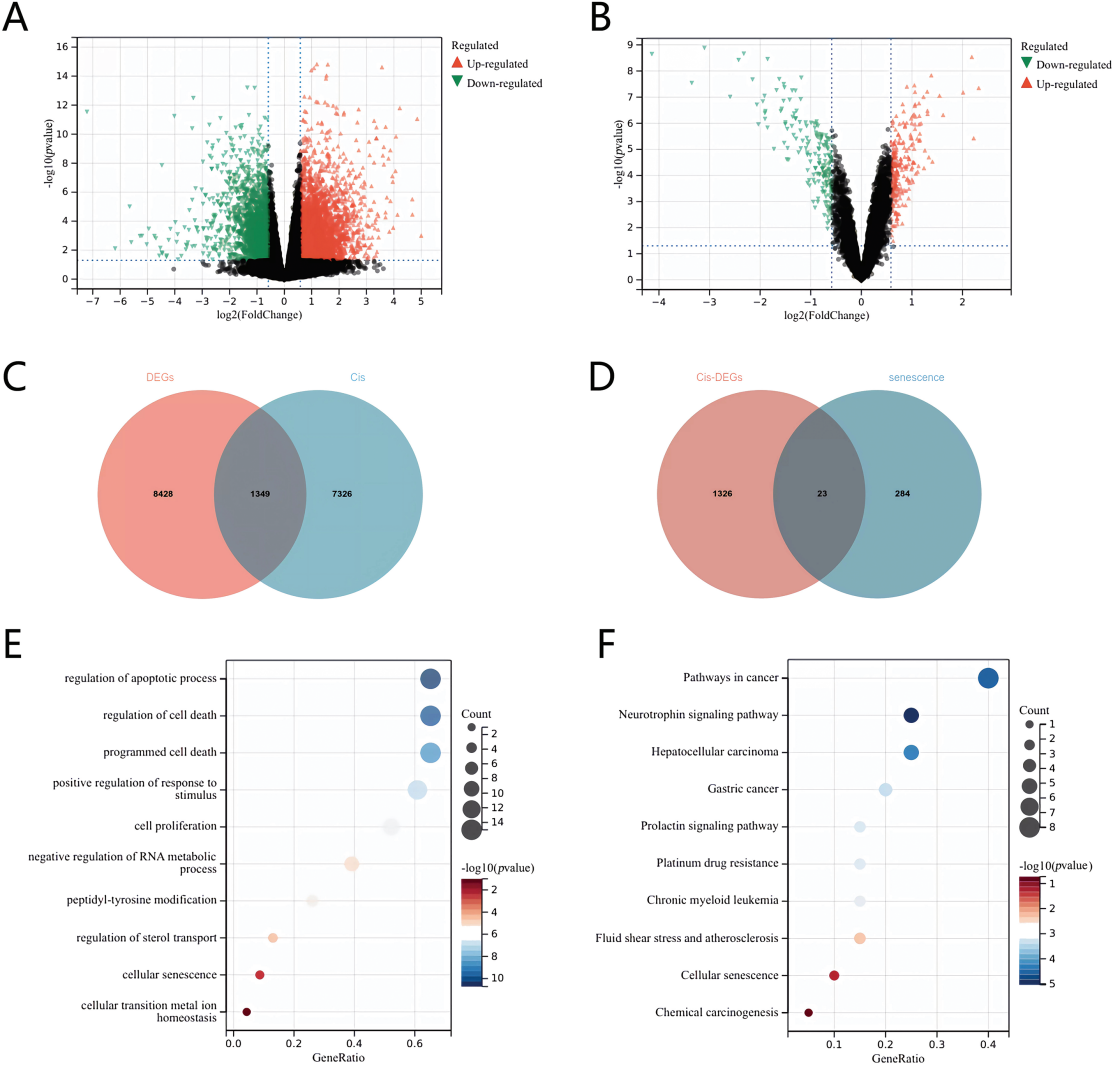
Screening of senescence differential genes associated with cisplatin sensitivity in neuroblastoma. (A) Volcano plot of differentially expressed genes in stage IV *vs*. other stages. (B) Volcano plot of genes linked to cisplatin sensitivity. (C) Venn diagram of differentially expressed genes and the cisplatin resistance dataset across stage IV and other stages. (D) Venn diagram of genes related to cisplatin sensitivity and aging. (E) GO enrichment analysis. (F) KEGG enrichment analysis.

We conducted an analysis of gene expression profiles and clinical data from 498 NB samples sourced from the GEO dataset (GSE49710). The samples were classified into high-risk (stage IV) and low-risk groups. Utilizing the Limma package, we applied a fold change threshold of 1.5 and a *p*-value threshold of <0.05, which enabled us to identify a total of 9777 genes with differential expression. Among these, 7062 genes were found to be upregulated, while 2715 genes were downregulated (Fig. 1A). To investigate genes associated with cisplatin resistance, we performed a comparison of the differentially expressed genes from stage IV NB against those from other disease stages as well as a cisplatin resistance dataset (GSE86842) using Venn diagram analysis, which revealed 1349 common genes (Fig. 1C). Further differential analysis of these shared genes (Fig. 1B) identified 23 genes that also overlapped with a set of 284 aging-related genes from The Ageing Gene Database, exhibiting differential expression in response to both cisplatin treatment and aging (Fig. 1D). Subsequently, we performed Gene Ontology (GO) functional enrichment analyses and Kyoto Encyclopedia of Genes and Genomes (KEGG) on the 23 identified genes. These analyses confirmed a significant association of these genes with the cellular senescence pathway in NB (Fig. 1E,F), suggesting a potential link between cellular senescence, cisplatin resistance, and the clinical outcomes in NB patients.

### Construction of a prognostic model using LASSO regression

**Figure 2:**
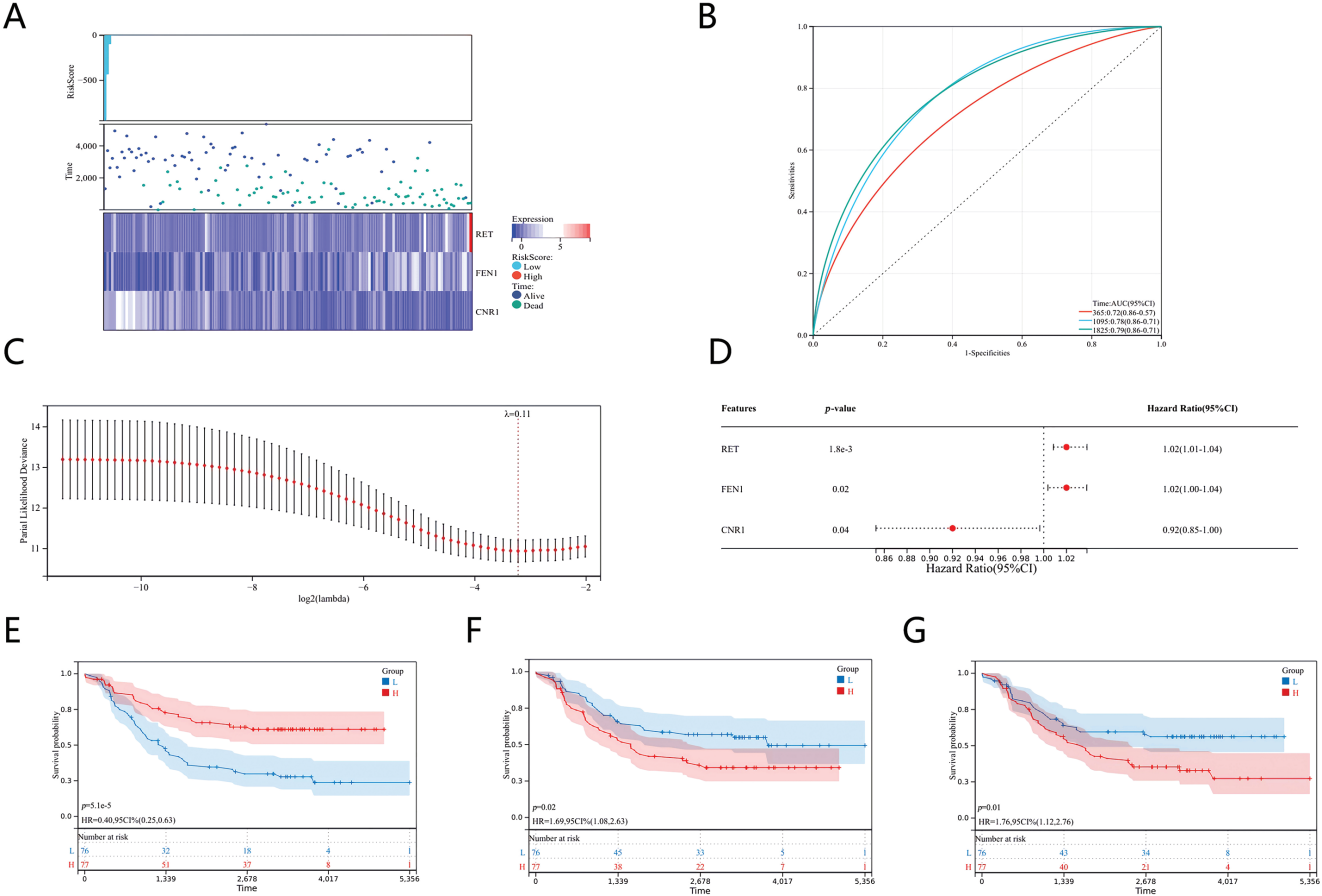
Prognostic models of genes associated with senescence and cisplatin sensitivity in neuroblastoma. (A) Heatmaps of differentially expressed genes linked to prognosis. (B) ROC curve analysis. (C) Coefficients of the selected features represented by lambda parameters. (D) Multivariate Cox regression analysis showing prognostic differences. (E) Kaplan-Meier survival curve for CNR1. (F) Kaplan-Meier survival curve for RET. (G) Kaplan-Meier survival curve for FEN1.

To develop a prognostic model for NB, we analyzed the relationships between various risk scores and identified three key genes—CNR1, RET, and FEN1—as potential prognostic indicators using the LASSO (Least Absolute Shrinkage and Selection Operator) algorithm (Fig. 2A).

We assessed the effectiveness of the prognostic model through ROC analysis using the pROC package in R software. The area under the curve (AUC) was calculated, along with calibration curves for 1, 3, and 5 years, employing the ci function in pROC to finalize the AUC and establish confidence intervals (Fig. 2B). The optimal prognostic risk model, derived from the LASSO algorithm with a λ value of 0.11, resulted in the following risk score formula: Risk Score = (−0.0504) * CNR1 + (0.0083) * RET + (0.00298) * FEN1. This formula quantifies risk based on the expression levels of the identified genes (Fig. 2C). To further validate this model, we conducted survival analysis using the “survival” package in R, assessing the prognostic impact of the 23 characteristics linked to NB. Multivariate Cox proportional hazards regression analysis was performed to identify prognostic disparities among the genes (Fig. 2D). Our findings indicated that CNR1 is associated with a more favorable prognosis in NB, whereas RET and FEN1 are correlated with a poorer prognosis. These associations are illustrated in the Kaplan-Meier survival curves for each gene (Fig. 2E–G), supporting the clinical relevance of the identified genes in predicting patient outcomes.

### FEN1 is a key prognostic gene related to cisplatin sensitivity in neuroblastoma

**Figure 3:**
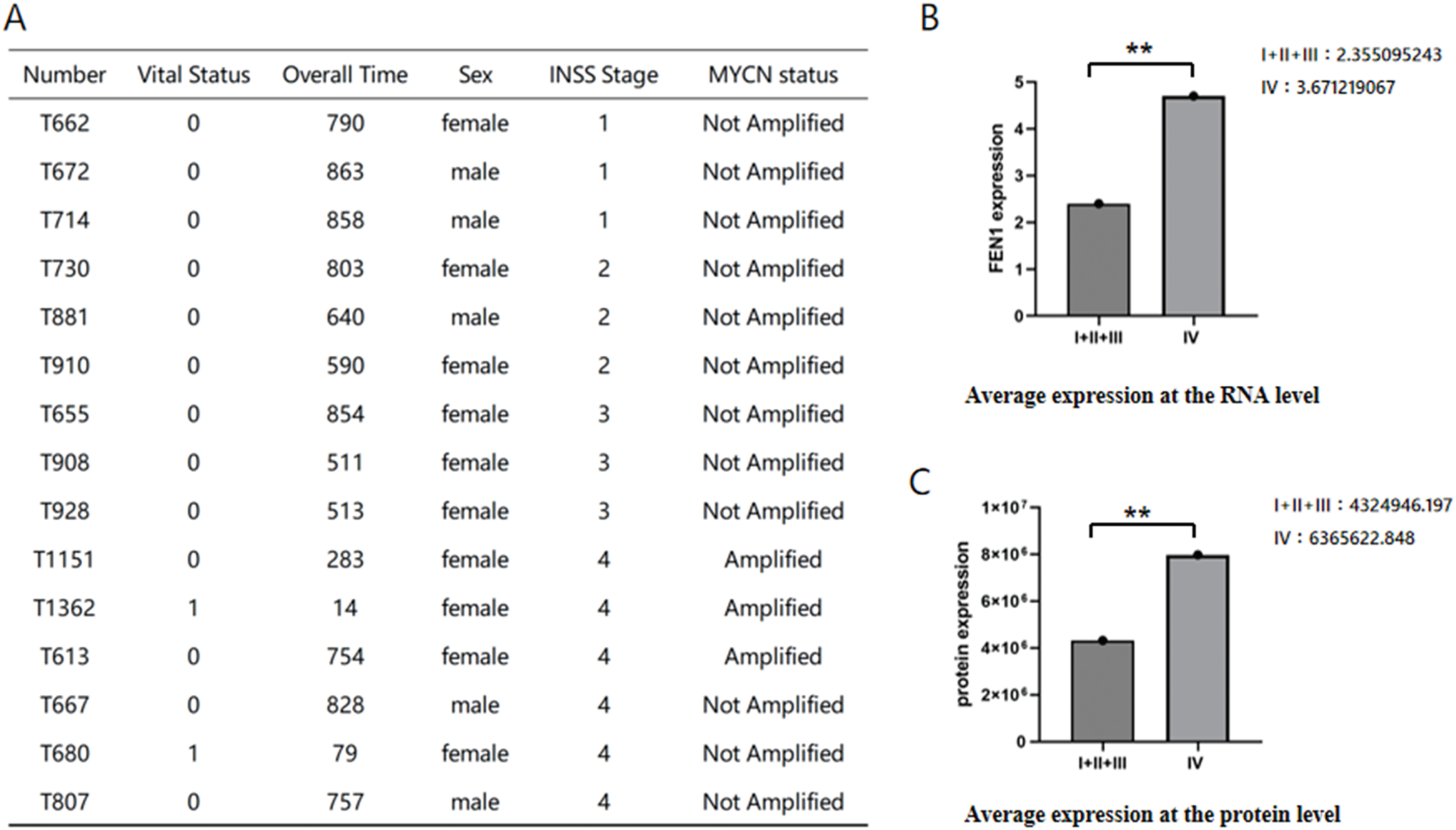
Differential expression of FEN1 in NB across stages based on sequencing data. (A) Clinical information for the 15 patients whose samples were sequenced. (B) Average differential expression of FEN1 at the RNA level between stage IV and other stages. (C) Average differential expression of FEN1 at the protein level between stage IV and other stages (**p < 0.01).

We conducted an analysis of 15 NB samples obtained from the Children’s Hospital of Fudan University, consisting of 5 male and 10 female patients, with ages ranging from 18 days to 16 years and 8 months. Among these patients, 3 exhibited MYCN amplification, while the remaining patients did not. The disease staging revealed that 6 patients had stage IV disease, while the other 9 patients were categorized as having stage I, II, or III disease. Notably, all stage IV patients who received chemotherapy are still alive (Fig. 3A). Transcriptomic and proteomic sequencing was performed on these samples to gain insights into gene expression and protein profiles. The analysis focused on three previously identified genes—CNR1, RET, and FEN1—that play important roles in NB survival. The results indicated that only FEN1 was significantly upregulated in stage IV compared to the other stages (Fig. 3B,C). This finding underscores the critical role of FEN1 as a gene associated with poor prognosis and sensitivity to cisplatin in NB, suggesting its potential as a target for therapeutic intervention and further studies into its mechanisms of action in NB.

### Correlation between FEN1 expression and cisplatin IC50 in NB cell lines

**Figure 4:**
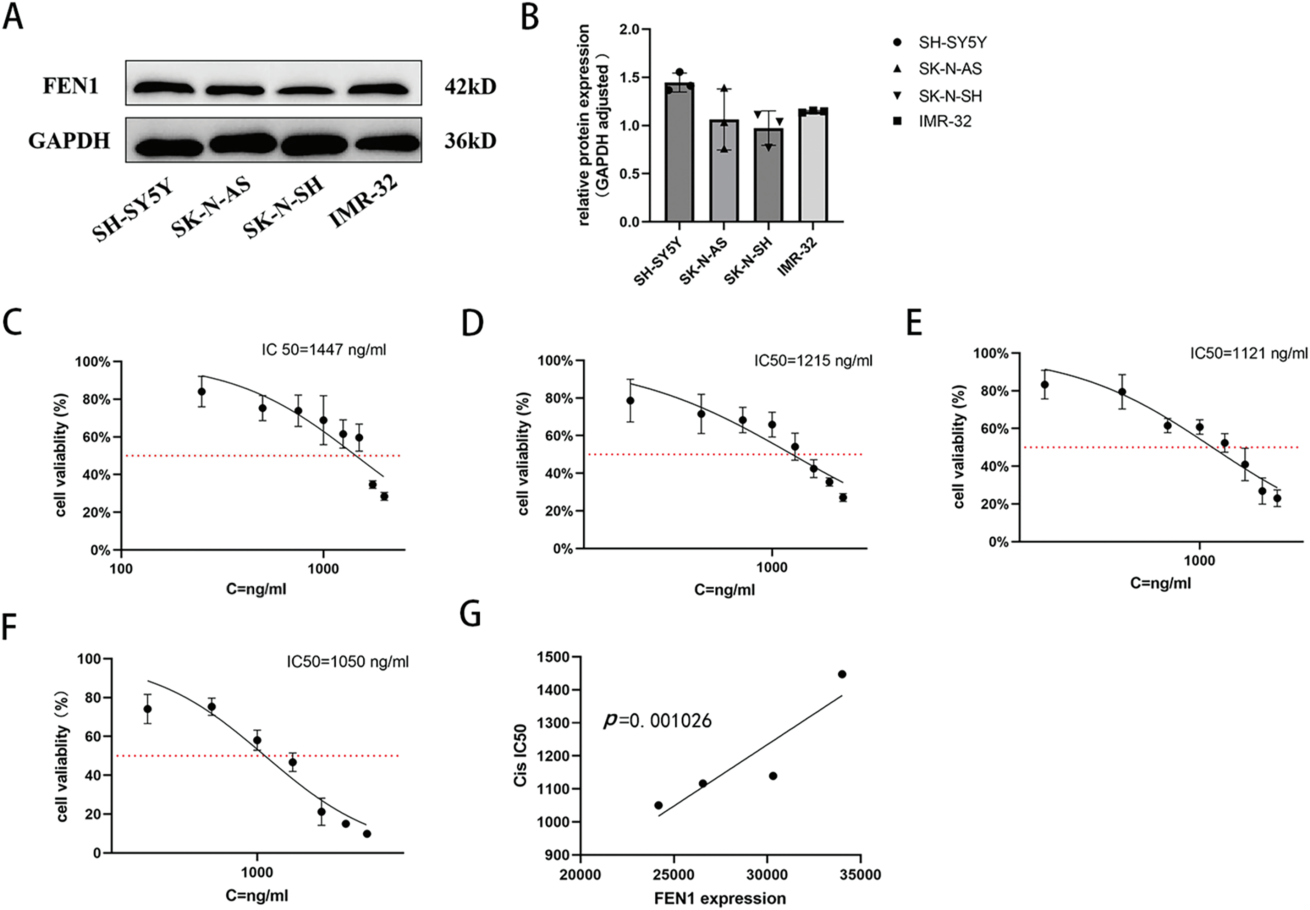
FEN1 expression in different NB cell lines was positively correlated with cisplatin IC50. (A) Western blot analysis of FEN1 expression in different neuroblastoma cell lines. (B) Histogram showing the relative expression levels of FEN1. (C) Cisplatin IC50 values for the SH-SY5Y cell line. (D) Cisplatin IC50 values for the IMR-32 cell line. (E) Cisplatin IC50 values for the SK-N-AS cell line. (F) Cisplatin IC50 values for the SK-N-SH cell line. (G) Plot illustrating the correlation between FEN1 expression levels and cisplatin IC50 values across neuroblastoma cell lines (*p* < 0.01; n = 3).

We evaluated the basal expression levels of FEN1 across various NB cell lines using Western blot (WB) analysis. The results demonstrated that FEN1 expression was highest in the SH-SY5Y cell line and lowest in the SK-N-SH cell line (Fig. 4A,B). To assess the sensitivity of these cell lines to cisplatin, we treated cells from each line with increasing concentrations of cisplatin for 48 h, followed by a cell viability assay using the CCK-8 method. The calculated cisplatin IC50 values were as follows: 1447 ng/mL for the SH-SY5Y cell line (Fig. 4C), 1215 ng/mL for the IMR-32 cell line (Fig. 4D), 1121 ng/mL for the SK-N-AS cell line (Fig. 4E), and 1050 ng/mL for the SK-N-SH cell line (Fig. 4F). Further analysis indicated a positive correlation between the basal expression levels of FEN1 and the cisplatin IC50 values across the NB cell lines (Fig. 4G, *p* < 0.01). This suggests that higher levels of FEN1 expression are associated with increased resistance to cisplatin treatment, implicating FEN1 as a potential biomarker for cisplatin sensitivity in NB and highlighting its relevance in therapeutic approaches for this malignancy.

### Attenuating FEN1 mitigates proliferation, invasion, and migration of NB cells

**Figure 5:**
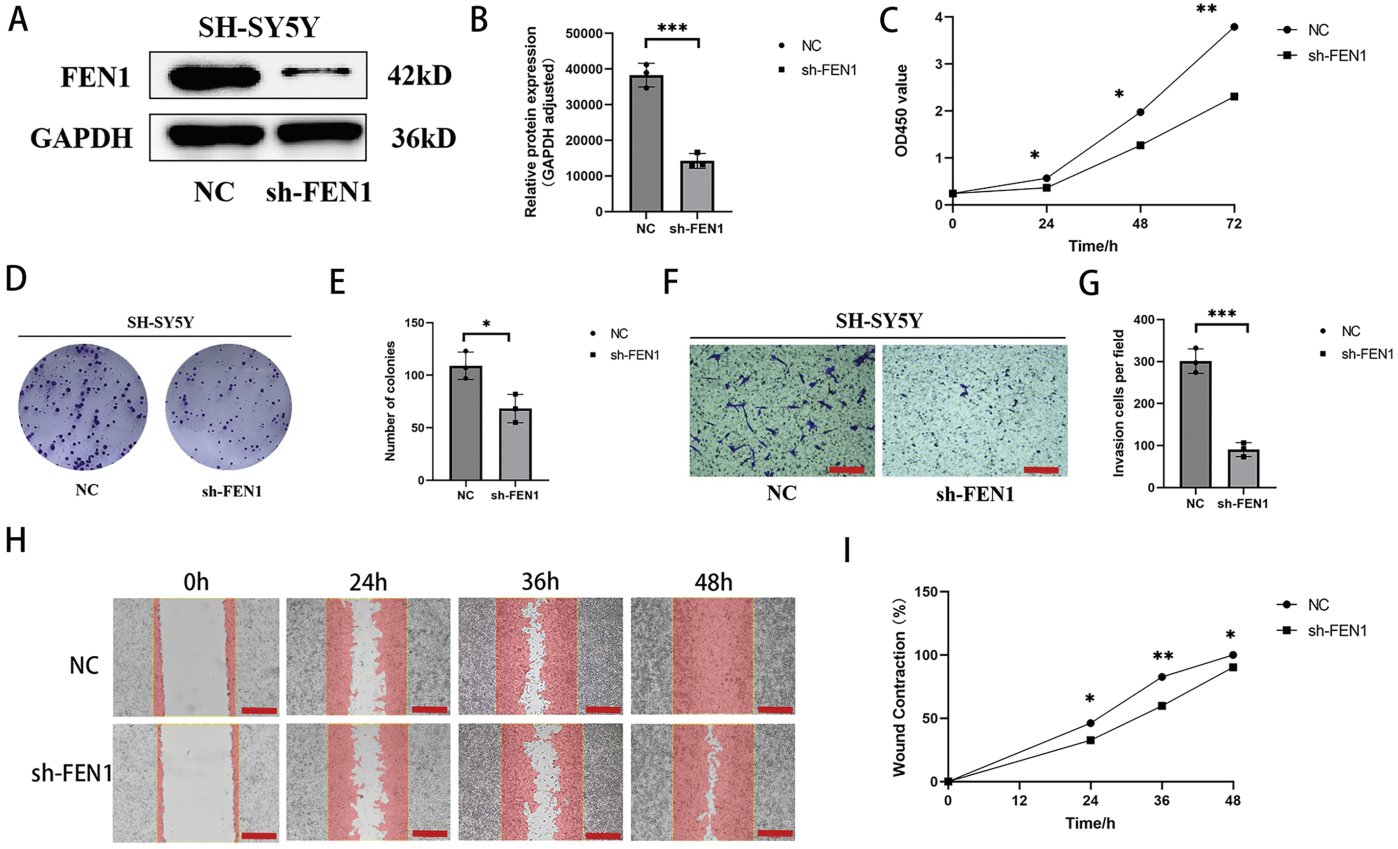
FEN1 knockdown can inhibit the proliferation, migration and invasion of NB cells. (A) Western blot analysis confirming FEN1 knockdown in the SH-SY5Y cell line. (B) Histogram showing FEN1 expression levels in SH-SY5Y cells. (C) CCK-8 assay results demonstrating proliferation of NB cells at 0, 24, 48, and 72 h after FEN1 knockdown. (D) Clonogenic assay illustrating colony formation post-FEN1 knockdown. (E) Statistical analysis of clonogenic assay results. (F) Transwell assay showing invasion of NB cells after FEN1 knockdown. (G) Statistical plot of Transwell assay results. (H) Scratch assay assessing migration of NB cells at 0, 24, 36, and 48 h post-FEN1 knockdown. (I) Statistical graph of scratch assay results (Transwell assay scale: 100 μm; Scratch assay scale: 500 μm; *p < 0.05; **p < 0.01; ***p < 0.001; n = 3).

FEN1 has been identified as a pro-tumor gene that promotes tumor progression in various cancer types; however, its role in NB had not been previously studied. In this study, we investigated the effects of FEN1 knockdown in the SH-SY5Y cell line, which exhibited the highest expression of FEN1. The efficiency of the knockdown was confirmed through Western blot (WB) analysis (Fig. 5A,B). Following FEN1 knockdown, we observed a significant reduction in the proliferation, invasion, and migration abilities of NB cells. Cell proliferation was assessed using the CCK-8 assay at multiple time points (0, 24, 48, and 72 h) post-FEN1 knockdown (Fig. 5C), revealing a marked decrease in cell proliferation at all assessed intervals. In addition, clonogenic assays indicated a reduced cloning efficiency in FEN1-knockdown cells (Fig. 5D,E), suggesting that the capacity for long-term survival and growth was compromised. To evaluate the effects of FEN1 knockdown on cell invasion and migration, we employed Transwell and scratch assays, respectively. The Transwell assay demonstrated a significant reduction in the number of invasive cells following FEN1 knockdown (Fig. 5F,G). Similarly, the migration assay revealed decreased migration rates at 0, 24, 36, and 48 h post-scratch (Fig. 5H,I). Collectively, these findings indicate that FEN1 knockdown impairs the proliferation, invasion, and migration capabilities of NB cells, highlighting its potential role as a therapeutic target in NB treatment.

### FEN1 overexpression promotes proliferation, invasion, and migration of NB cells

**Figure 6:**
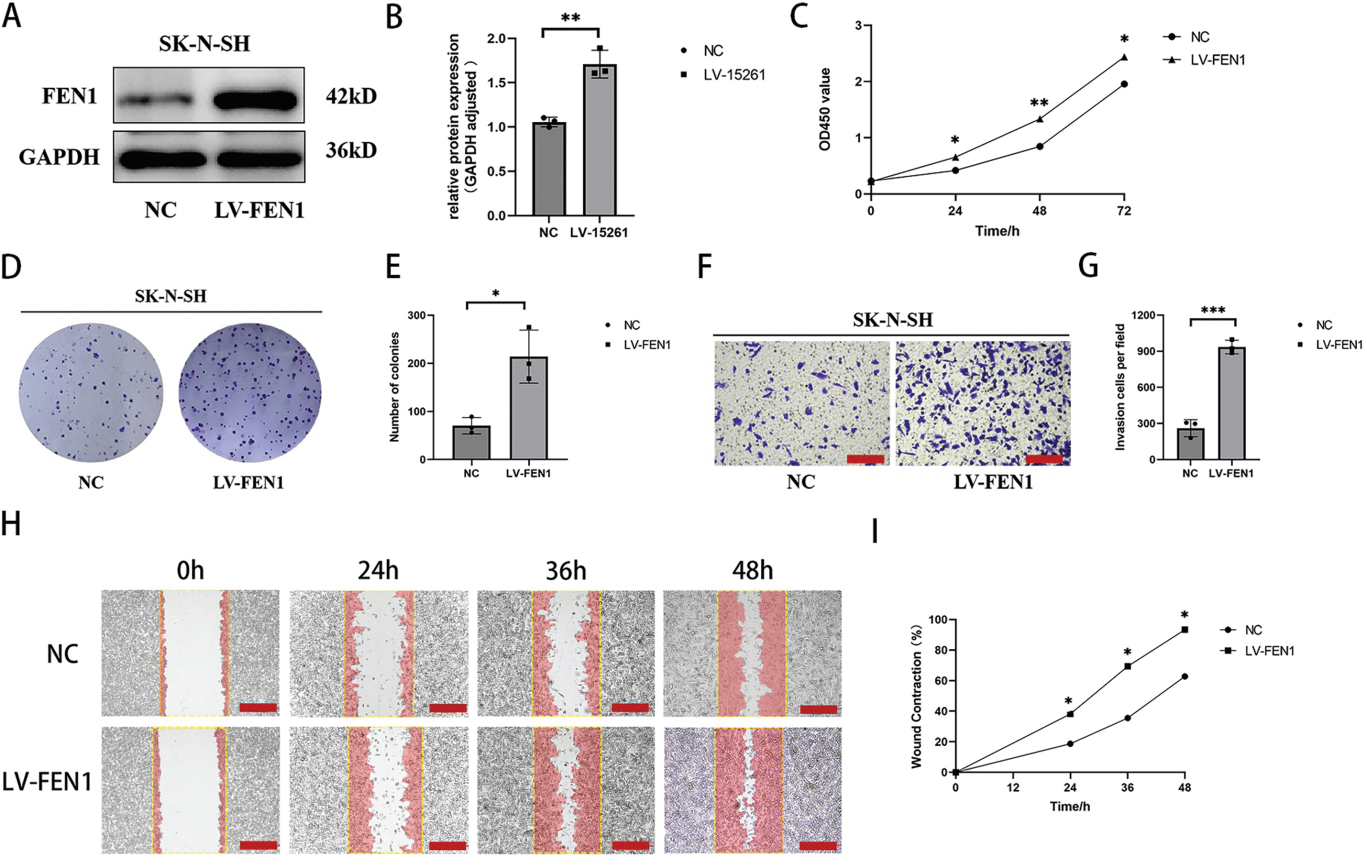
Overexpression of FEN1 promotes the proliferation, migration and invasion of NB cells. (A) Western blot analysis confirming FEN1 overexpression in the SK-N-SH cell line. (B) Histogram showing FEN1 expression levels in SK-N-SH cells. (C) CCK-8 assay results demonstrating proliferation of NB cells at 0, 24, 48, and 72 h after FEN1 overexpression. (D) Clonogenic assay illustrating colony formation post-FEN1 overexpression. (E) Statistical analysis of clonogenic assay results. (F) Transwell assay showing invasion of NB cells after FEN1 overexpression. (G) Statistical plot of Transwell assay results. (H) Scratch assay assessing migration of NB cells at 0, 24, 36, and 48 h post-FEN1 overexpression. (I) Statistical graph of scratch assay results (Transwell assay scale: 100 μm; Scratch assay scale: 500 μm; *p < 0.05; **p < 0.01; ***p < 0.001; n = 3).

To investigate the impact of FEN1 overexpression on NB cell function, we utilized the SK-N-SH cell line, which exhibits the lowest basal expression of FEN1. FEN1 overexpression was achieved through lentiviral transfection, and the success of this transfection was confirmed by Western blot (WB) analysis (Fig. 6A,B). Following FEN1 overexpression, we conducted several assays to assess changes in cell functions. The CCK-8 assay, performed at 0, 24, 48, and 72 h, demonstrated a significant increase in cell proliferation in response to FEN1 overexpression (Fig. 6C). Additionally, clonogenic assays illustrated enhanced colony formation in cells that overexpressed FEN1 (Fig. 6D,E), indicating an increased capacity for long-term growth. To evaluate the effects on invasion and migration, we employed Transwell and scratch assays, respectively. The Transwell assay showed a marked increase in the number of invasive cells following FEN1 overexpression (Fig. 6F,G). Similarly, the scratch assay revealed enhanced migration at 0, 24, 36, and 48 h post-scratch (Fig. 6H,I). These results collectively indicate that FEN1 overexpression significantly enhances the proliferation, invasion, and migration abilities of NB cells, aligning with the pro-cancer effects of FEN1 observed in various other tumor types. This underscores the potential role of FEN1 as a driver of tumor progression in NB, suggesting its importance as a target for therapeutic strategies.

### FEN1 modulates cisplatin sensitivity in NB by regulating cellular senescence

**Figure 7:**
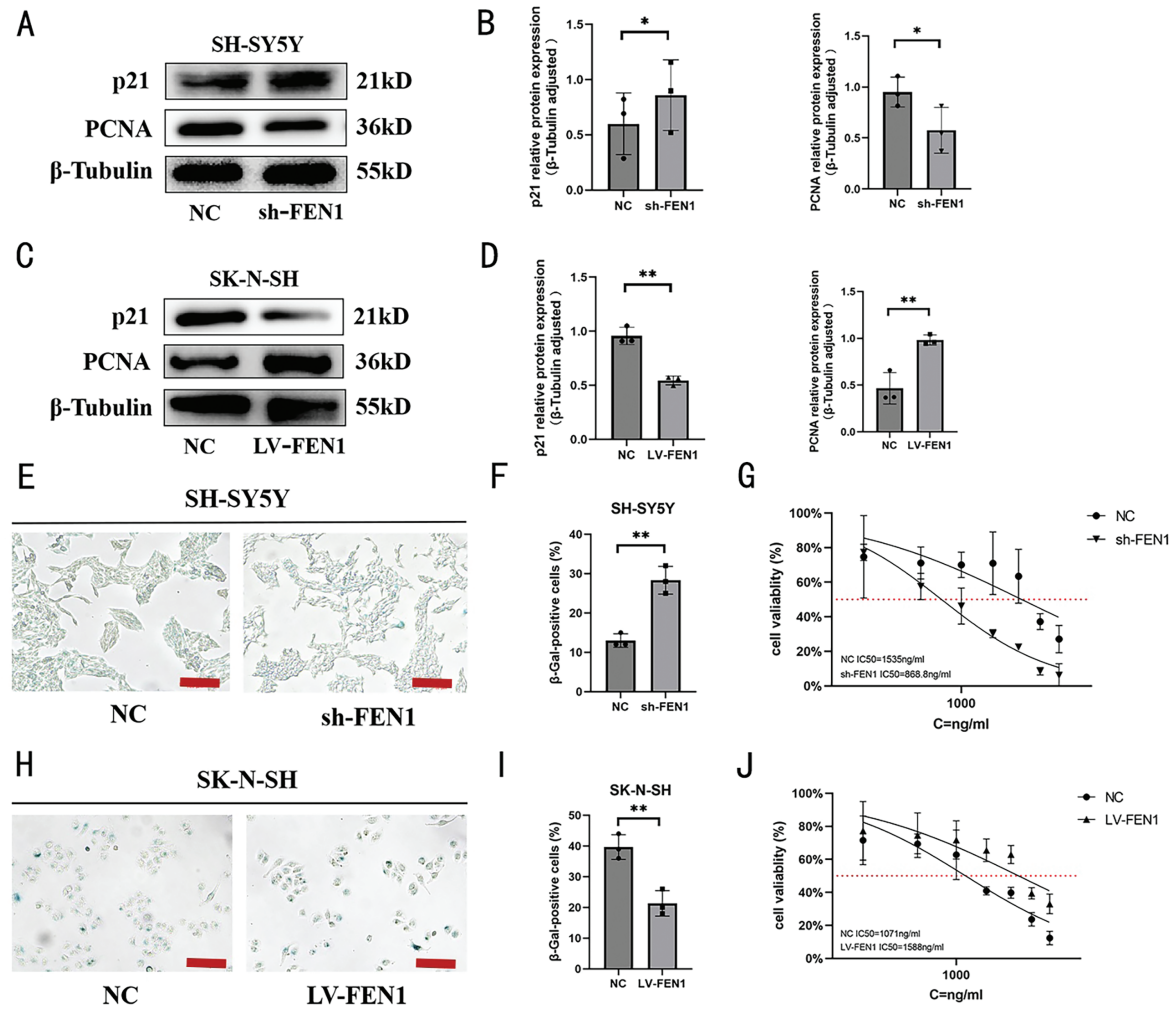
FEN1 modulates cisplatin sensitivity through cellular senescence regulation in NB. (A) Western blot analysis of senescence-associated proteins p21 and PCNA following FEN1 knockdown in SH-SY5Y cells. (B) Histogram of p21 and PCNA expression levels in SH-SY5Y cells post-FEN1 knockdown. (C) Western blot analysis of p21 and PCNA expression following FEN1 overexpression in SK-N-SH cells. (D) Histogram of p21 and PCNA expression levels in SK-N-SH cells post-FEN1 overexpression. (E) β-Galactosidase staining of senescent NB cells following FEN1 knockdown in SH-SY5Y cells. (F) Histogram showing the percentage of senescent cells in SH-SY5Y cells post-FEN1 knockdown. (G) Cisplatin IC50 values in SH-SY5Y cells following FEN1 knockdown. (H) β-Galactosidase staining of senescent NB cells following FEN1 overexpression in SK-N-SH cells. (I) Histogram showing the percentage of senescent cells in SK-N-SH cells post-FEN1 overexpression. (J) Cisplatin IC50 values in SK-N-SH cells following FEN1 overexpression (β-Galactosidase staining assay scale: 100 μm; *p < 0.05; **p < 0.01; n = 3).

Recent literature points to p21, a cyclin-dependent kinase inhibitor, as playing a crucial role in cellular senescence, influencing a variety of processes including apoptosis, differentiation, and migration [28]. Proliferating cell nuclear antigen (PCNA), which functions as a DNA polymerase accessory protein, is also recognized as a significant marker for cellular senescence [29]. Furthermore, β-galactosidase (β-gal) is well-established as a biomarker for identifying senescent cells in mammalian tissues [30]. To explore the role of FEN1 in regulating cisplatin sensitivity through cellular senescence, we first assessed the expression of senescence-related proteins p21 and PCNA in SH-SY5Y cells with FEN1 knockdown via Western blot (WB) analysis (Fig. 7A). We observed an increase in p21 expression coupled with a decrease in PCNA expression following FEN1 knockdown (Fig. 7B). Additionally, we performed β-galactosidase staining to evaluate the population of senescent cells. The results revealed a significant increase in the number of senescent cells post-FEN1 knockdown (Fig. 7E,F). These findings collectively suggest that FEN1 may play a pivotal role in modulating cellular senescence, which in turn could impact the sensitivity of NB cells to cisplatin treatment. The increase in p21 and the decrease in PCNA, along with enhanced β-galactosidase staining, indicate that FEN1 knockdown promotes a senescent phenotype in SH-SY5Y cells, potentially contributing to altered drug sensitivity and highlighting its significance in NB therapy.

In contrast, in SK-N-SH cells that overexpress FEN1, Western blot analysis demonstrated decreased expression of p21 and increased expression of PCNA (Fig. 7C,D). Additionally, β-galactosidase staining indicated a reduction in the proportion of senescent cells in these overexpressing cells (Fig. 7H,I). To further evaluate the impact of FEN1 on cisplatin sensitivity, we measured the IC50 values of cisplatin under both conditions of FEN1 knockdown and overexpression. The results showed that knockdown of FEN1 led to a decreased cisplatin IC50, indicating increased sensitivity to the drug (Fig. 7G). Conversely, FEN1 overexpression resulted in an increased cisplatin IC50, which reflects reduced sensitivity to cisplatin (Fig. 7J). These findings collectively suggest that FEN1 plays a significant role in modulating cisplatin sensitivity in NB cells through its influence on cellular senescence. Specifically, FEN1 knockdown enhances sensitivities to cisplatin by promoting senescence, while its overexpression decreases sensitivity, potentially by suppressing senescence markers. This underscores the potential of targeting FEN1 in therapeutic strategies to enhance the effectiveness of cisplatin in NB treatment.

### Cisplatin and FEN1 inhibitor C20 significantly enhances NB cell proliferation inhibition

**Figure 8:**
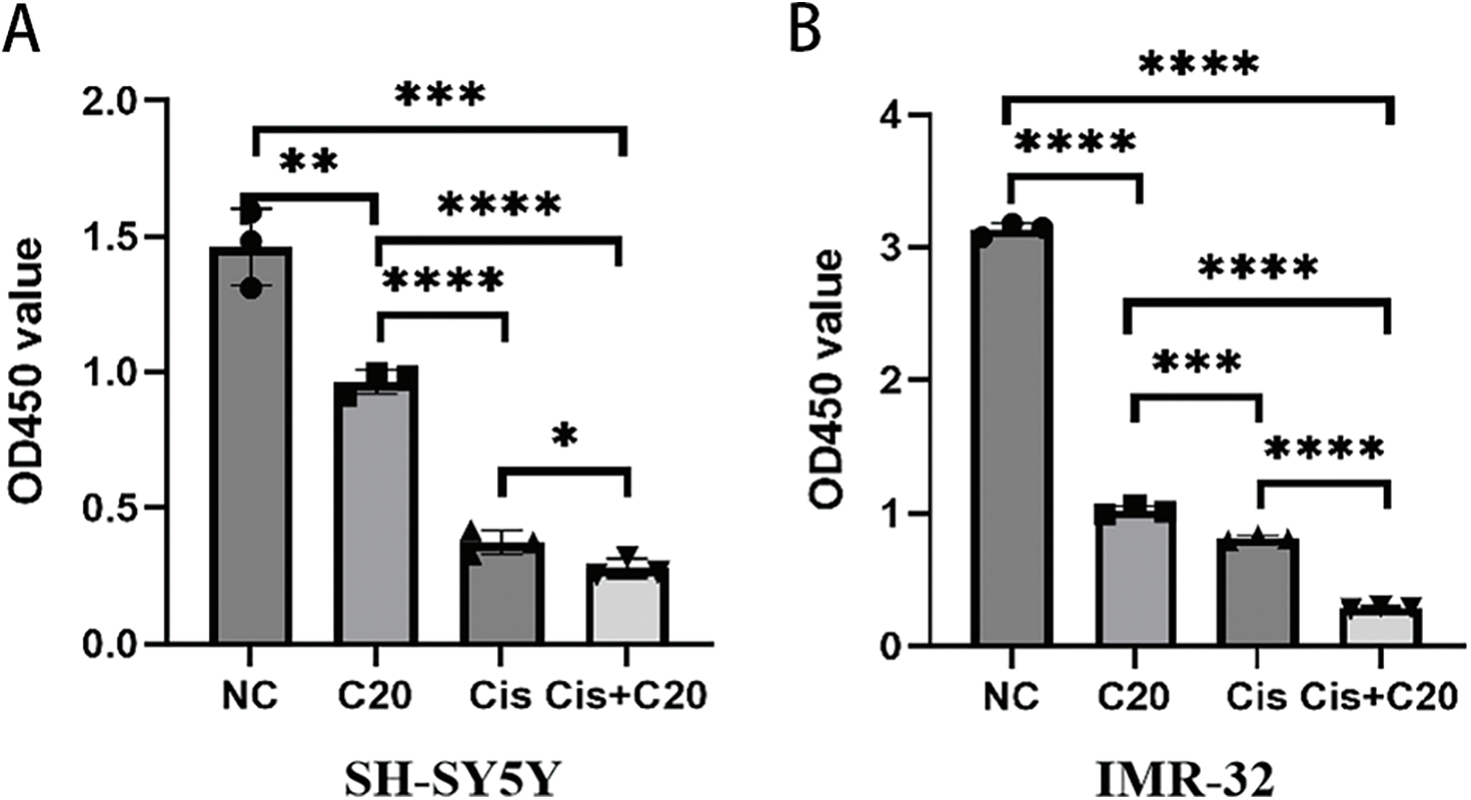
FEN1 inhibitors can enhance the antitumor effect of cisplatin in neuroblastoma. (A) CCK-8 proliferation assay in SH-SY5Y cells showing the effects of cisplatin, FEN1 inhibitor C20, and their combination. (B) CCK-8 proliferation assay in IMR-32 cells showing the effects of cisplatin, FEN1 inhibitor C20, and their combination (*p < 0.05; **p < 0.01; ***p < 0.001; ****p < 0.0001; n = 3).

Finally, we assessed the effects of combining cisplatin with the FEN1 small molecule inhibitor C20 on two NB cell lines, SH-SY5Y and IMR-32. The results indicated that treatment with 5 μM C20 for 24 h alone led to a significant reduction in cell viability for the SH-SY5Y cell line when compared to the untreated control group. Moreover, the combination of 1500 ng/mL cisplatin and 5 μM C20 for 24 h resulted in a significantly greater inhibition of cell survival than 1500 ng/mL cisplatin treatment alone (Fig. 8A). Similar findings were observed in the IMR-32 cell line, where the combined treatment of 1500 ng/mL cisplatin and 5 μM C20 for 24 h also exhibited a markedly greater reduction in cell survival compared to 1500 ng/mL cisplatin administered as a monotherapy (Fig. 8B). Overall, these results suggest that C20 enhances the cytotoxic effects of cisplatin on NB cells, indicating its potential as a therapeutic agent in improving the efficacy of cisplatin treatment. This combination strategy could provide a promising approach to enhance the treatment outcomes for patients with NB.

### Discussions

The five-year survival rates for NB vary significantly among various risk groups, with rates exceeding 90% for low-risk patients, while those categorized as high-risk have survival rates dipping below 50% [31]. strategies for NB are predominantly influenced by biological risk factors [32]. Surgical intervention stands as the primary method for treating low-risk patients. Conversely, high-risk individuals generally require a comprehensive treatment strategy that combines high-dose chemotherapy, surgery, radiotherapy, and monoclonal antibody therapy [33]. Cisplatin, frequently utilized alone or in conjunction with other chemotherapeutic agents such as cyclophosphamide, doxorubicin, or etoposide [34,35], faces considerable challenges regarding clinical efficacy due to chemoresistance. The issue of reduced drug sensitivity in tumor therapy necessitates an investigation into the mechanisms that affect chemotherapeutic sensitivity. This research merges bioinformatics analyses of extensive NB datasets with gene sequencing of clinical samples to pinpoint the gene FEN1. This gene, associated with age-related processes, demonstrates a significant correlation with both poor prognosis and cisplatin sensitivity in NB. The findings highlight the critical need to understand the role of FEN1 in the progression of NB and its potential as a target for mitigating chemoresistance.

FEN1 is a crucial valvular endonuclease involved in DNA repair, and its overexpression has been associated with tumor progression and heightened cell proliferation [36]. For instance, in gastric cancer, FEN1 levels are higher when compared to normal tissue, with elevated expression correlating to poor prognoses for patients [37]. A study conducted by Simon Keane et al. in 2022 found that FEN1 expression is significantly greater in NB tissues compared to adjacent non-tumorous tissues [38]. Our own bioinformatics analyses and sequencing data corroborate these findings, demonstrating that FEN1 expression is markedly elevated in stage IV NB compared to earlier stages, suggesting its potential as a prognostic indicator for adverse outcomes in this cancer. Additionally, functional assays performed on NB cells reveal that FEN1 promotes cell proliferation, migration, and invasion, indicating its involvement in oncogenic processes within NB. This gene has been extensively studied as an age-related factor in multiple contexts, including gastric cancer [26], pancreatic cancer [27], and chronic obstructive pulmonary disease (COPD) [28]. It has been linked to the regulation of pivotal cellular pathways, such as the G2/M phase transition [39], the MYC pathway [40], the p53 pathway [41], and autophagy [42], all of which are associated with cellular senescence [43]. The induction of cellular senescence by DNA damage highlights FEN1’s critical role in DNA repair, reinforcing its status as a significant gene related to senescence [44]. Given its importance in maintaining the stability of double-stranded DNA structures, FEN1’s involvement in cellular senescence is both plausible and noteworthy. In this study, we further investigate FEN1’s role as a central senescence gene in NB through bioinformatics analysis, emphasizing its potential as a therapeutic target.

Cellular senescence serves as a essential tumor-suppressing mechanism, characterized by chromatin remodeling, metabolic reprogramming, and elevated senescence-associated β-galactosidase (SA-β-gal) activity, all of which significantly contribute to tumorigenesis and progression [45]. The process is initiated by p21, an essential regulator of senescence [46]. Senescent cells exhibit growth arrest and lack proliferation markers, particularly PCNA, which is widely recognized as a senescence marker [6]. Our study explored the role of FEN1 in cellular senescence within NB cells. Utilizing SA-β-gal staining, we assessed changes in senescence following the modulation of FEN1 in the SH-SY5Y and SK-N-SH cell lines. Upon knocking down FEN1 in SH-SY5Y cells, we observed an increase in p21 levels and a decrease in PCNA, thereby enhancing markers of senescence. Conversely, the overexpressing FEN1 in SK-N-SH cells resulted in reduced p21 levels and elevated PCNA, subsequently diminishing senescence markers. These findings underscore the dual role of FEN1 in regulating both tumor progression and senescence in NB cells.

In addition to its role in inhibiting suppressing tumor cell progression, cellular senescence is also intricately associated with the sensitivity of NB cells to cisplatin chemotherapy. Despite its significance, the influence of cellular senescence on NB progression and cisplatin sensitivity remains inadequately explored. In our study, we assessed the cisplatin IC50 values of NB cells following the knockdown or overexpression of FEN1. Our results indicated that an increase in the population of senescent cells, induced by FEN1 knockdown, led to a significant enhancement in cisplatin sensitivity. Conversely, FEN1 overexpression correlated with a reduced number of senescent cells and a substantial decrease in cisplatin sensitivity. These findings are consistent with existing research and suggest that FEN1 influences cisplatin sensitivity through its effects on cellular senescence, providing critical insights for clinical treatment strategies.

Chemotherapy agents induce DNA damage in tumor cells, and their efficacy can be augmented when used in conjunction with inhibitors that target DNA damage repair pathways [47]. FEN1 has emerged as a promising target for such inhibitors, with prior studies indicating that inhibiting FEN1 can enhance the efficacy of chemotherapy drugs known to induce DNA damage [48], including cisplatin [49] and 5-fluorouracil [50]. Furthermore, research conducted by Li et al. demonstrated that FEN1 inhibitors can enhance the sensitivity of cervical cancer cells to radiotherapy [51], while Mesquita et al. found that FEN1 inhibitors increase cisplatin sensitivity in ovarian cancer cells [15]. The primary mechanism of FEN1 inhibitors involves disrupting DNA damage repair mechanisms, consequently impairing FEN1’s ability to repair damaged DNA. In our study, we examined the effects of combining cisplatin with the FEN1 inhibitor C20 in the SH-SY5Y and IMR-32 cell lines. The results revealed that this combination not only effectively killed NB cells but also amplified the cytotoxic effects of cisplatin, positioning FEN1 as a promising anti-cancer target and underscoring the potential of its inhibitors to enhance treatment outcomes for NB.

## Conclusion

In conclusion, our study identifies FEN1 as a pivotal prognostic marker in NB that carries negative implications. FEN1 plays a significant role in regulating NB cell proliferation, migration, and invasion. Its inhibition leads to cellular senescence and enhances sensitivity to cisplatin, offering valuable insights into the mechanisms underlying NB resistance. Furthermore, FEN1 inhibitors not only hinder NB cell proliferation but also enhance the chemotherapeutic effectiveness of cisplatin, paving the way for novel strategies in the clinical management of high-risk NB patients. However, this study has not been conducted in animal experiments or clinical trials, and the specific mechanisms involved still require further exploration on our part.

## Data Availability

All data generated for this manuscript have been included in this article. The publicly available data are provided in GEO databases.

## List of Abbreviations

NB: Neuroblastoma
SA-β-gal: Senescence-associated β galactosidase
TIS: Therapy-induced senescence
CDK: Cyclin-dependent kinases
FEN1: Flap endonuclease-1
CCK-8: Cell counting kit-8
TCGA: The cancer genome atlas
SASP: Senescence-associated secretory phenotype
HSP90B1: Heat Shock Protein 90 Beta Family Member 1
LASSO: Least Absolute Shrinkage and Selection Operator
PCNA: Proliferating Cell Nuclear Antigen
BER: Base excision repair
HRR: Homologous Recombination Repair
NHEJ: Non-homologous End Joining
COPD: Chronic obstructive pulmonary disease
PBS: Phosphate buffer saline
INSS: International Neuroblastoma Staging System
GSEA: Gene Set Enrichment Analysis
KEGG: Kyoto Encyclopedia of Genes and Genomes
GO: Gene Ontology
CNR1: Cannabinoid Receptor 1
GEO: Gene Expression Omnibus
ROC: Receiver Operating Characteristic
AUC: Area Under Curve
IC50: 50% inhibitory concentration
WB: Western blot
OD: Optical density
PVDF: Polyvinylidene difluoride
